# Sudden-Onset Sensorineural Hearing Loss and Tinnitus in a Patient With Rheumatoid Arthritis: A Case Report and Literature Review

**DOI:** 10.7759/cureus.38739

**Published:** 2023-05-08

**Authors:** Beatrice E Torere, Swetha Chittipolu, Gabriel Alugba, Henry O Aiwuyo, Jennifer L Kennard

**Affiliations:** 1 Internal Medicine, North Mississippi Medical Center, Tupelo, USA; 2 Internal Medicine, Englewood Hospital and Medical Center, Englewood, USA; 3 Internal Medicine, One Brooklyn Health/Brookdale Hospital Medical Center, New York City, USA; 4 Rheumatology, North Mississippi Health Services, Tupelo, USA

**Keywords:** hearing impairment, autoimmune, literature review, case report, rheumatoid arthritis, tinnitus, sensorineural hearing loss

## Abstract

Rheumatoid arthritis (RA) can affect the auditory system either as a direct complication of the disease course or secondary to medication adverse effects. Rheumatoid arthritis-induced autoimmune inner ear disease can present as tinnitus, conductive hearing loss, sensorineural hearing loss (SNHL), or mixed. According to previously published articles, SNHL is the most common hearing loss in RA. Age, smoking, noise exposure, and alcohol may affect the disease progression. Here, we present a case of a 79-year-old female who presented to the rheumatology clinic with complaints of abrupt onset bilateral hearing loss with associated tinnitus; pure tone audiometry confirmed sensorineural hearing loss. Her tinnitus resolved completely, and her hearing improved significantly after treatment with steroids and leflunomide. Based on this case and previous literature, we conclude that rheumatoid arthritis is the cause of SNHL in our patient. Appropriate and timely medical interventions have been reported to improve the prognosis of hearing impairment in rheumatoid arthritis patients. Our case highlights the need to have a high index of suspicion of rheumatoid arthritis-induced autoimmune inner ear disease in an elderly patient presenting with sudden-onset hearing impairment and the importance of prompt referral to a rheumatologist.

## Introduction

Rheumatoid arthritis (RA) is an autoimmune inflammatory disease that affects multiple organs such as the heart, lungs, skin, and eye in addition to primary joint manifestations [1]. The auditory system can be affected by various pathologies along the disease course. Sensorineural hearing loss (SNHL) is the most common type of hearing impairment (HI) seen in patients with RA, noting a prevalence of 25%-75%, followed by conductive (CHL) and mixed hearing loss (MHL) [2]. Autoimmune sensorineural hearing loss is well responsive to corticosteroids. Autoimmune sensorineural hearing loss typically presents with an idiopathic, rapidly progressive, predominantly bilateral sensorineural hearing loss, and it responds well to corticosteroid therapy.

The pathophysiology of hearing impairment in RA is not well known. However, some findings show relations to arthritis of incudostapedial and incudomalleolar joints resulting in conductive hearing loss or deposition of the immune complex, hence sensorineural hearing loss [3]. Other etiologies have been linked to drugs such as salicylates, non-steroidal anti-inflammatory drugs, and disease-modifying antirheumatic drugs. More so, environmental factors, such as smoking, alcohol, and exposure to noise, have been shown to affect the auditory system in healthy individuals and patients with RA [3,4].

Diagnosis is based on clinical findings, and different types of hearing tests are available; pure tone audiometry (PTA) from 125 to 8,000 Hz is mostly used for evaluating hearing loss (HL) [5]. Extended high-frequency audiometry, which tests very high frequencies ranging from 10,000 to 20,000 Hz, is a useful test for identifying hearing loss early on before it affects medium and low frequencies that significantly alter the hearing capacity [5,6].

Treatment for RA-induced autoimmune inner ear disease is high-dose glucocorticoids (60 to 80 mg prednisone) every morning for two to three weeks and commencement or escalation of disease-modifying antirheumatic drugs (DMARDs) [7]. This usually leads to remarkable hearing improvement or complete hearing recovery. Steroids are then tapered gradually until hearing loss returns, thereby establishing the correct chronic steroid dose [7,8].

## Case presentation

The patient is a 79-year-old female with a past medical history of normal pressure hydrocephalus (status post-ventriculoperitoneal shunt placement in the 1980s), nicotine dependence, and 40 years history of meningioma. She was referred by the otolaryngologist to our rheumatology clinic after being diagnosed with SNHL and positive rheumatoid factor (RF). 

**Figure 1:**
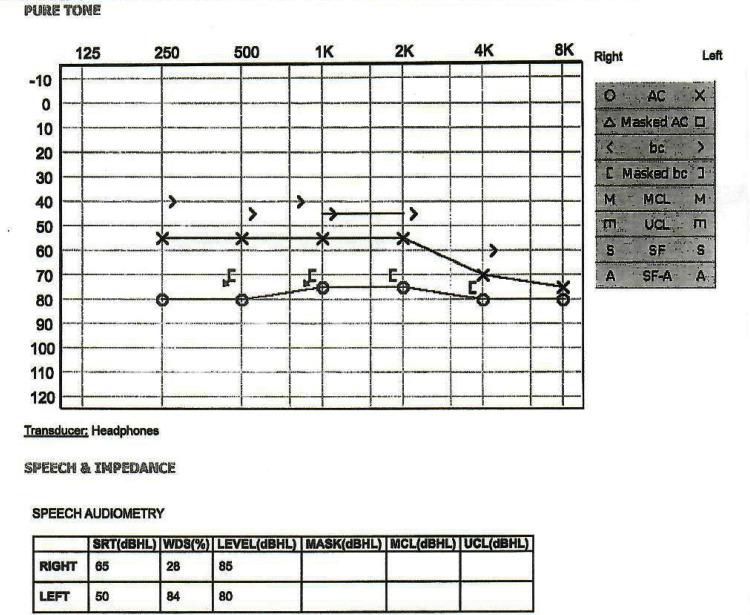
Audiometry threshold assessment showing moderately severe to severe sensorineural hearing loss (SNHL) of the left ear and severe SNHL of the right ear AC: air conduction, bc: bone conduction, O or triangle: result from the right ear measured with headphones (air conduction), X or a square: result from the left ear measured with headphones (air conduction), S: information is not ear-specific, < or [: the result from the right ear measured with bone conduction, > or ]: the result from your left ear measured with bone conduction, M:  Most comfortable loudness level (MCL), m: uncomfortable listening level (UCL), S: threshold recorded in the sound field (SF) that cannot be assigned to either ear, A: aided sound field (SF-A), SRT: speech recognition threshold, WDS: Word Discrimination Score, dB: loudness in decibels, Hz: frequency in Hertz.

Four months before her rheumatology clinic presentation, the patient developed sudden-onset unilateral hearing loss. She stated that she woke up one morning and could not hear from her right ear. Within one week, her symptoms progressed to bilaterally hearing loss with associated tinnitus. She was evaluated by an otolaryngologist and underwent pure tone audiometry (See Figure 1). Her evaluation was remarkable for bilateral sensorineural hearing loss, positive rheumatoid factor, and elevated acute phase reactants.

**Figure 2:**
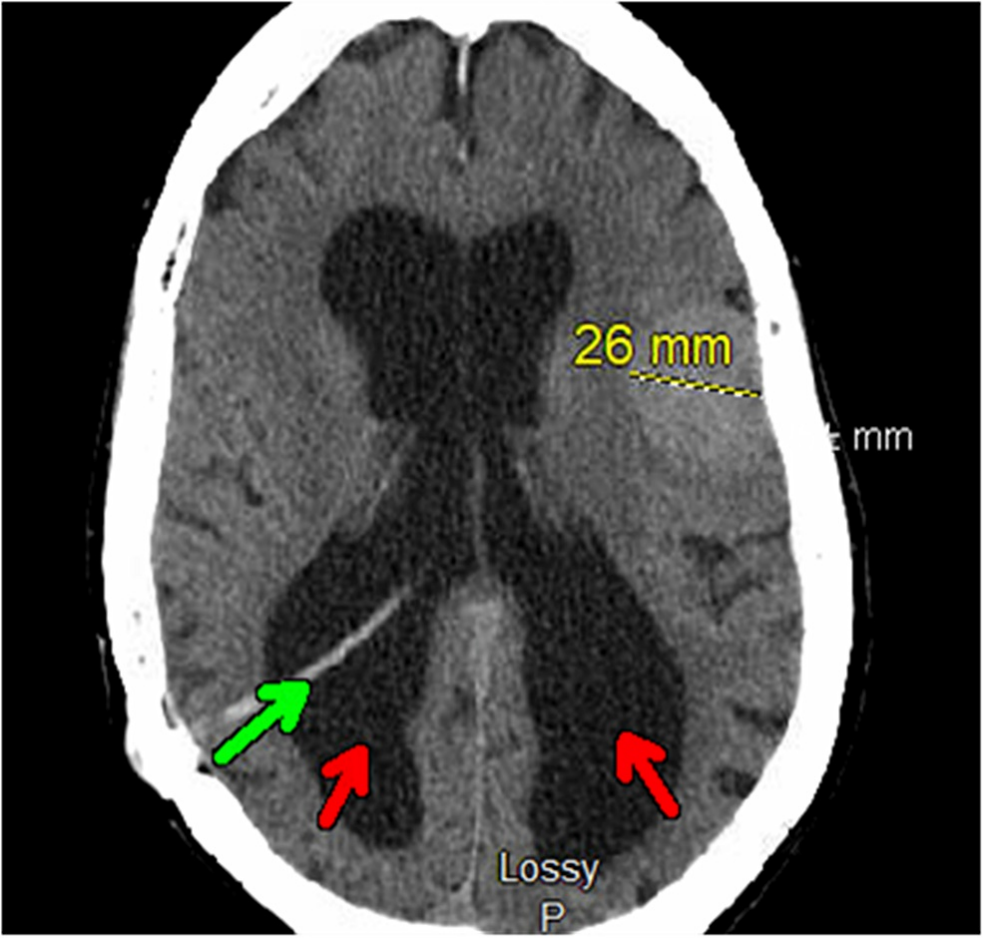
Axial view: computed tomography of the head without contrast Axial view: computed tomography of the head without contrast shows a stable 2.6 cm left frontal lobe meningioma with associated local mass effect. The green arrow indicates a right transparietal approach ventriculostomy shunt, and the red arrows indicate persistent ventriculomegaly.

Computed tomography (CT) of the head without contrast revealed a 2.6 cm left frontal lobe meningioma with associated local mass effect, a right transparietal approach ventriculostomy shunt, and persistent ventriculomegaly (stable chronic findings compared to her previous head imaging) (Figure 2).

**Figure 3:**
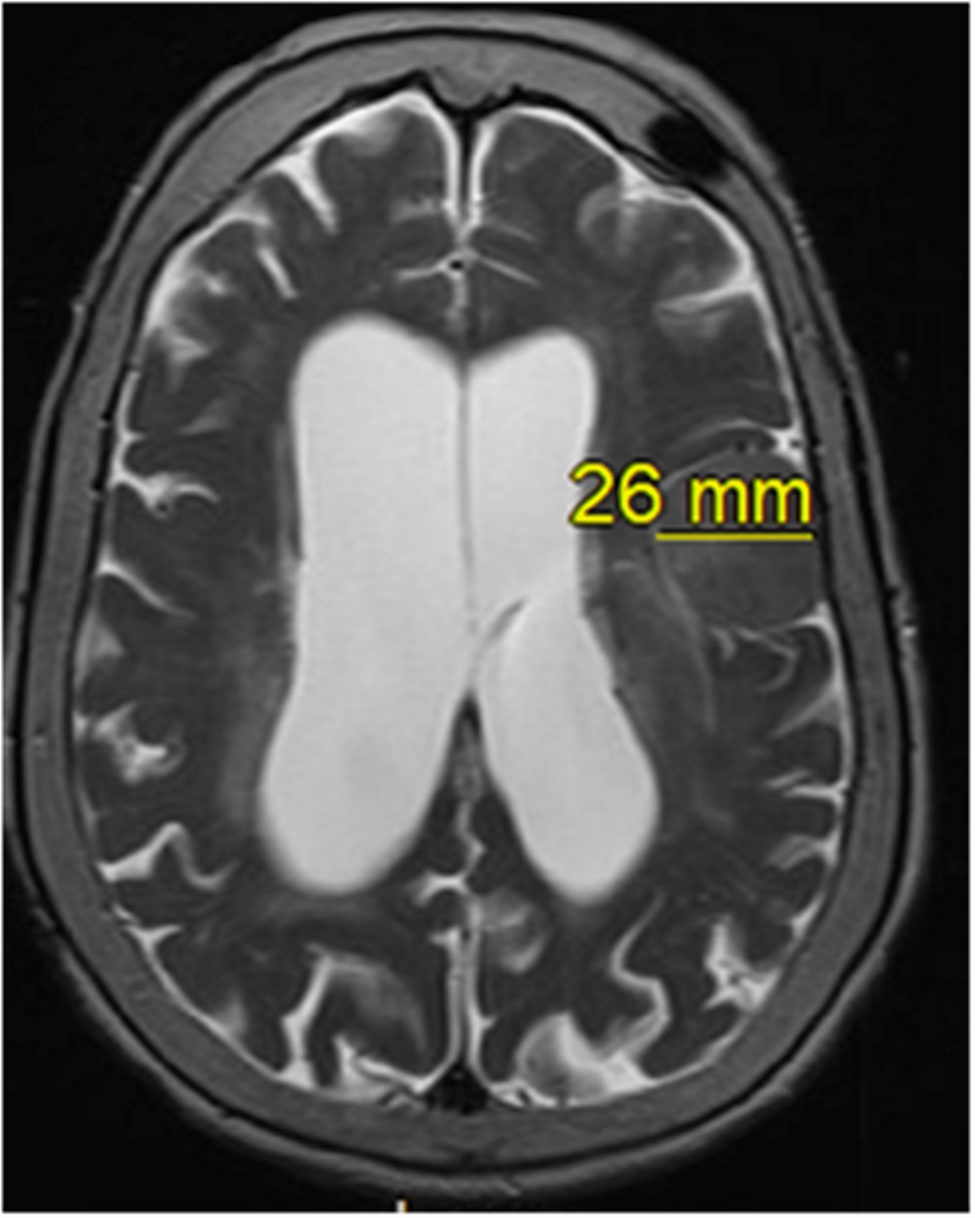
Axial view MRI of the brain without contrast Axial view T2 (transverse relaxation time) showing chronic, stable 2.6 cm meningioma compared to previous MRI.

**Figure 4:**
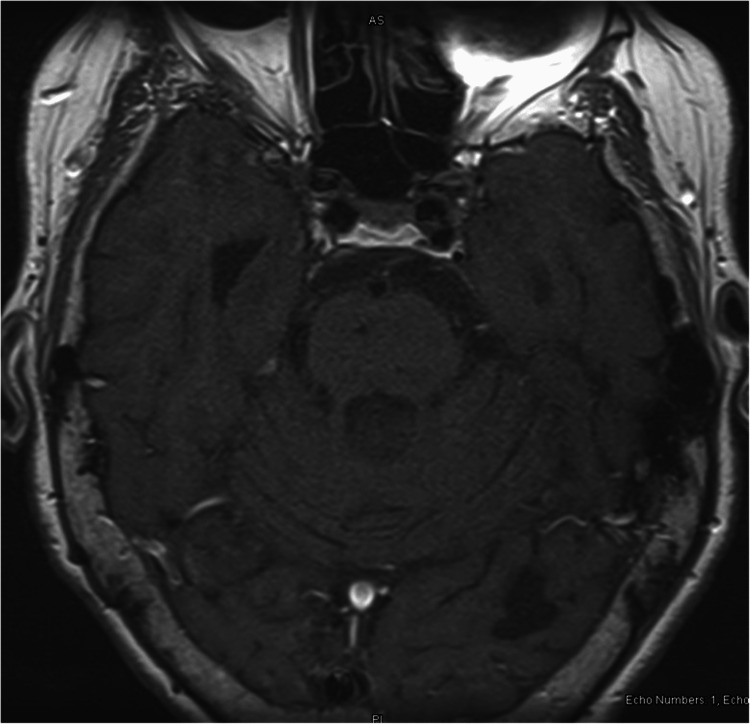
Axial view of MRI brain without contrast Axial T1 (longitudinal relaxation time) showing no enhancing retrocochlear mass lesion.

Magnetic resonance imaging (MRI) revealed no acute intracranial abnormality and no enhancing retrocochlear mass lesion (Figures 3, 4).

At the rheumatology clinic, the patient complained of bilateral hearing loss, tinnitus, and joint pains in her hands and feet. She also reported morning stiffness in her hands lasting less than 30 minutes daily. She denied joint swelling, ear pain, discharge, recent head trauma, nasal congestion, sore throat, fever, and chills. She takes no medication. 

Upon chart review by the rheumatologist, it was noted that the patient was diagnosed with rheumatoid arthritis at an outside facility 11 years before the index presentation. At that time (11 years ago), she complained of bilateral ankle pain, and her investigation was remarkable for +RF IgM > 100, +RF IgG 7.5, +RF IgA 20, +CCP >300, and erythrocyte sedimentation rate (ESR) 17 and C-reactive protein (CRP) 0.5. Computed tomography of the ankle was reported as osteoarthritis (OA). There was no referral letter to a rheumatologist. Laboratory findings at the outside facility 11 years before the index presentation are shown in Table 1.

**Table 1:** Remarkable laboratory findings 11 years prior to index presentation

Test	Findings	Reference range
Rheumatoid factor (IU/mL)	Positive; IgM >100 and IgG 7.5, IgA 20	<15
Anti-cyclic citrullinated peptide (Units)	Positive; >300	<20
Erythrocyte sedimentation rate (mm/hr)	17	0-30
C-reactive protein (mg/dl)	0.5	<0.9

**Figure 5:**
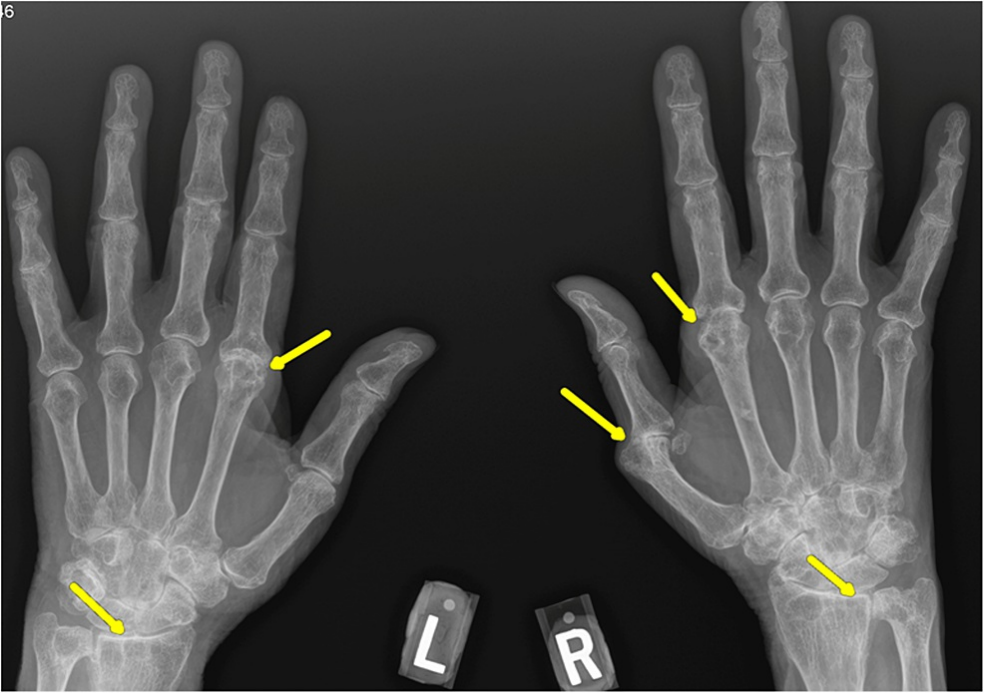
Anterior/posterior view of hand X-ray Yellow arrows indicate significant joint space narrowing and findings of both rheumatoid arthritis and osteoarthritis.

Physical examination of her auricular cartilages, ear canals, and tympanic membranes showed no erythema, swelling, or tenderness. Ear canals were patent and intact. The whisper test revealed significantly reduced hearing in the left ear and no hearing in the right ear; normal oral aperture; no oral ulcerations and no skin ulcerations or pits on fingertips; and squaring over the bilateral carpometacarpal joint, no joint tenderness, erythema, and swelling. Anterior/posterior view of the hand X-ray revealed significant joint space narrowing and findings of both rheumatoid arthritis and osteoarthritis (Figure 5). Laboratory findings at the rheumatology clinic visit are shown in Table 2.

**Table 2:** Remarkable laboratory findings at the rheumatology clinic visit AI: antibody index.

Test	Findings	Reference
Rheumatoid factor (IU/mL)	44	<15
Cyclic citrullinated peptide IgG Antibodies (units)	>250	<20
Erythrocyte sedimentation rate (mm/hr)	46	0-30
C-reactive protein (mg/dl)	0.9	<1.0
White blood cell count (×1,000 UL)	4.05	5.0-10.0
Hemoglobin (mg/dl)	13.9	12.0-16.0
Platelet count (×1,000)	243	
Antinuclear antibody	Negative	Negative
Myeloperoxidase antibody (AI)	<1.0	<1.0
Anti-neutrophilic cytoplasmic antibody	Negative	Negative
Proteinase 3 antibody (AI)	<1.0	<1.0
Hepatitis C virus antibody	Negative	Negative
Hepatitis B core antibody	Negative	Negative
Hepatitis B surface antigen	Negative	Negative

The patient received counseling on smoking cessation and was started on prednisone 60 mg daily. Her hearing impairment significantly improved after four weeks of steroid therapy. Prednisone was gradually tapered, and she was started on a steroid-sparing agent/DMARD, leflunomide.

## Discussion

Rheumatoid arthritis (RA) is a chronic inflammatory condition that primarily affects the synovium causing destructive arthritis [9]. However, RA is known to have extra-articular manifestations (EAMs) involving the lungs, heart, eyes, skin, ears, and other body structures. Sensorineural hearing loss (SNHL) is the most prevalent form of hearing impairment in rheumatoid arthritis, with a prevalence rate between 12% and 80% [4]. Studies have reported that SNHL affects high frequency, although middle and low frequencies have also been seen [10,11]. SNHL has been associated with the duration of RA [12-14], disease activity, positive RF, and elevated acute phase reactants [12,15,16]. Distortion product otoacoustic emissions (DPOAEs) testing is used to assess the function of the cochlear hair cells as a possible origin of SNHL, and this test evaluates the function of the cochlear outer hair cells [17]. Studies have revealed that patients with RA have increased rates of abnormal DPOAEs compared to healthy controls [18,19].

A pathologic condition that damages the cochlea and/or cochlear nerve results in sensorineural hearing loss (SNHL) [20]. Several mechanisms have been proposed linking RA to SNHL, of which autoimmune damage or immune complex deposition in the cochlea hair cells is a predominantly mentioned mechanism; other proposed mechanisms include vasculitis of ‘vasa vasorum’, nerve neuritis, and ototoxicity from medications used to treat RA (steroids, nonsteroidal anti-inflammatory drugs (NSAIDs), disease-modifying antirheumatic drugs) [3,20,21].

As an autoimmune condition, RA is linked to adaptive immune system malfunction, which leads to the development of various autoantibodies [22,23]. The autoantibody-antigen reactions or cytotoxic actions of these autoantibodies may directly damage the cochlear hair cells causing SNHL [20]. Rheumatoid vasculitis is a well-known consequence of RA that is more frequently observed in seropositive patients with severe, long-standing disease and is seen to affect any blood vessels of any organ [24]. In rheumatoid vasculitis, immune complex deposition in the labyrinthine artery may result in dire complications in the cochlear, considering that the cochlea has minimal collateral circulation. Thus, even a slight disruption to blood supply can result in hair cell injury, stria vascularis atrophy, and spiral ganglion degeneration, thereby leading to SNHL [20].

SNHL in RA could also be caused by retrocochlear involvement because immunological complexes that are formed in the endothelium of the vasa vasorum, which supplies the cochlear nerve, may cause auditory neuropathy [24]. Medications used in the treatment of RA have been shown to play a role in the damage of the cochlea or cochlear nerve. Studies have shown salicylates, NSAIDs antimalarial, and some disease-modifying antirheumatic drugs to cause SNHL due to their effect on the cochlear hair cells [3]. Hydroxychloroquine, an antimalarial medication routinely used in the treatment of RA, is associated with damage to the cochlear hair cells and other supporting sensory structures in the inner ear with atrophy of the stria vascularis leading to SNHL [25,26]. Biological DMARDs such as etoricoxib and abatacept are known as ototoxic agents, increasing the risk for SNHL in RA patients [27]. Several studies have demonstrated that ototoxicity from antirheumatic medications contributes marginally to SNHL seen in patients with RA [20]. However, our patient was not on any medication when she presented to the rheumatology clinic.

Lifestyle and environmental factors are synergistically associated with SNHL in patients with RA. Elderly patients with RA are known to be prone to hearing impairment [10], and nicotine intake has been shown to increase the risk for SHNL in RA patients, as it causes vasoconstriction and subsequent decrease in oxygen supply leading to damage of the hair cells and decline in cochlear function [3]. Our 79-year-old patient also had a history of nicotine dependence which may have contributed to SNHL. Long-term alcohol intake may play a role in SNHL in patients with RA due to its harmful effects on the outer cochlear hair cells [28].

Our patient thought her symptoms were due to her history of meningioma and normal pressure hydrocephalus; however, magnetic resonance imaging of her brain showed no enhancing retrocochlear mass lesion. Her symptoms improved following the commencement of prednisone medication, suggesting that the cause could have been most likely autoantibody damage to the hair cells or from immune complex deposition on the cochlear hair cells. This case highlights the need to have a high index of suspicion of rheumatoid arthritis-induced autoimmune inner ear disease in elderly patients presenting with sudden-onset hearing impairment and the importance of prompt referral to a rheumatologist.

## Conclusions

While it is primarily known that there are many potential causes and multiple contributors to the pathogenesis of sensorineural hearing loss in patients with rheumatoid arthritis, the temporal association of hearing impairment without other strong evidence favors rheumatoid arthritis as the culprit of SNHL in our patient. Using ototoxic medications for different purposes and the effects of DMARDs could be potential confounders; however, there was no history of prior use in our patient. We also highlight the variability in steroid response to autoimmune SNHL and the need to consider steroid-sparing agents as they could be beneficial. Several studies have shown improved outcomes with appropriate and timely effective medical intervention.
